# Supplementary material for: Acute kidney injury among critically ill neonates in a tertiary hospital in Tanzania; Prevalence, risk factors and outcome
Source: PLoS One. 2020 Feb 13;15(2):e0229074. doi: 10.1371/journal.pone.0229074 (PMC7018051; doi:10.1371/journal.pone.0229074)
Supplement: Attachment — Submitted filename: Response to Reviewers.docx [file pone.0229074.s002.docx]

**Response to Reviewer #1 comments**

**MAJOR POINTS:**

To strengthen the introduction, the second paragraph could include what the average international estimated prevalence of AKI is and what is known about neonatal AKI in developing countries with similar limited facilities. Alternatively, the authors may indicate how neonatal rates of mortality from asphyxia, prematurity and sepsis differ in Tanzania compared to global outcomes to provide better context for the manuscript findings.

**-A statement has been added to include the average international estimated prevalence of AKI.**

Page 4, Line 76-78: “All critically ill neonates admitted in neonatal ward presenting with convulsion, altered level of consciousness, inability to feed, vomiting everything, respiratory distress, jaundice, hyper/hypothermia, dehydration and shock were eligible for this study.” Is it necessary to specify all diagnosis included if the only those with obvious congenital abnormalities of GU tract were excluded? Were there other diagnoses that were excluded?

**-This has been changed as recommended, and the work critically ill neonates has been used and the list of other diagnoses omitted.**

Page 4, Line 82-83: “Sample size was estimated using prevalence of AKI (33.3%) reported among admitted 83 neonates in Zimbabwe by Matyanga et al. [12]” Explain how this reference was used to determine sample size for this cross-sectional study.

**-Explanation of how sample size was obtained has been provided.**

Please specify if there were any missing data especially surrounding creatinine.

**-Serum creatinine on day 14 was performed for participants with AKI only, therefore participants without AKI have no values for creatinine for day 14.**

Please specify whether AKI definition was comparing just baseline + 48 hours after admission creatinine in the methods. In general, KDIGO guidelines recommend serum creatinine rise 1.5X baseline within 7 days but creatinine at 7 days did not seem to be included in the study.

- **The diagnosis of AKI was established by measuring serum creatinine at baseline and 48 hours after admission. This has been included in the methods section**

There also seems to be missing data regarding participants who got KUB Ultrasounds (74+14(died) = 88, but 119 had AKI).

**Ultrasound were performed in**

Indicate in results that no patients were on dialysis.

**-This has been indicated in the results section.**

Please specify how neonatal sepsis is defined? Should it be neonatal bacteremia instead? In generally, neonatal sepsis could occur from pneumonia, meningitis, etc. Where those with severe pneumonia and meningitis also considered to have neonatal sepsis?

**-Sepsis was used for neonates with severe infection with no defined focus of infection to differentiate with pneumonia and meningitis**

Specify whether team focused on only in-hospital death.

**-This study focused on in-hospital deaths only**

Page 15, Line 210-214: “The diagnosis of AKI was established by measuring serum creatinine at baseline and 48 hours after admission” should be included in the methods.

**-This has been added in the methods section as recommended.**

- The authors may want to highlight the amount of congenital abnormalities observed among those with AKI in abstract detected via ultrasound and how it relates to global rates in the discussion.

**-This has been included in the abstract as recommended**

Fig 1: Please indicate the distribution of AKI stages

**-Indicated as recommended**

Fig 2: Consider cleaning up the legend and consider 3 vertical box + whiskers plots to display the distribution of baseline, 72 hour and 14 day creatinine values for all patients to present the data more succinctly.

**-Changes made as indicated and box plot included in the results section**

The discussion seems to indicate what is consistent in this study with prior data but it would be better if it could also highlight what is different regarding these findings compared to prior findings and reasons for the differences.

**-The contrast in findings with other studies has been included in the discussion section as recommended.**

The authors may consider adding the following: A large limitation of this study is that the Muhimbili National Hospital appears to be a secondary/tertiary referral hospital, which likely would lead to overestimation of the prevalence of neonatal acute kidney injury.

**-Added as recommended**

**MINOR POINTS:**

Page 3, Line 52: 0 by 25 Initiative is to eliminate preventable deaths from AKI worldwide by 2025.

**-This has been amended**

Minor inconsistencies regarding whether MNH is a secondary or tertiary referral hospital.

**-This has been clarified**

Page 7, Line 137-138: Consider: “A total of 378 neonates were enrolled in this study, of which 81% (306/378) were ≤ 7 days in age and 59.8% were male (226/378).”

**-Changes made as recommended**

Page 7, Line 139: Consider: “Majority of the participants were full term (78.0%), had birth weight above 2500 grams (74.3%) and a five-minute Apgar score above 7 (82.5%).”

**-Changes made as recommended**

Table 2: define RDS and HIE

**-RDS and HIE defined**

Page 11, Line 189-191: Table 4 describes the outcome of study participants, the overall mortality in this study was 22.5% (85/378), and participants with AKI had significantly higher mortality (70.6%) as compared to those without AKI (29.4%) p value of 0.000◊ P- value should be < 0.01?

**-Amended as recommended**

Page 12, line 204- Missing period

**-Period added**

Page 13, Line 224 and 225: missing comma

**-Comma added**

Page 13, Line 229: clinicians

**-Changed**

Page 14, Line 228: missing period

**-Period added**
